# Essential oil from *Artemisia gmelinii* extenuates cellular integrity of *Sporothrix globosa* and its biofilms by steering intracellular ROS and basal ergosterol levels

**DOI:** 10.3389/ffunb.2026.1842749

**Published:** 2026-06-22

**Authors:** Acharya Balkrishna, Manisha Kabdwal, Monali Joshi, Yash Varshney, Meenu Tomer, Pardeep Nain, Savita Lochab, Anurag Varshney

**Affiliations:** 1 Drug Discovery and Development Division, Patanjali Research Foundation, Haridwar, Uttarakhand, India; 2 Department of Allied and Applied Sciences, University of Patanjali, Patanjali Yog Peeth, Haridwar, Uttarakhand, India; 3 Patanjali Yog Peeth (UK) Trust, Glasgow, United Kingdom; 4 Department of Microbiology, Drug Discovery and Development Division, Patanjali Research Foundation, Haridwar, Uttarakhand, India; 5 Department of Chemistry, Drug Discovery and Development Division, Patanjali Research Foundation, Haridwar, Uttarakhand, India

**Keywords:** anti-biofilm action, *Artemisia gmelinii*, essential oil (EO), *Sporothrix globosa*, sporotrichosis

## Abstract

**Aim:**

Sporotrichosis is a fungal zoonotic infection caused by the pathogenic clade of the *Sporothrix* species complex. Considered a neglected tropical disease, sporotrichosis now has a cosmopolitan distribution through species like *Sporothrix globosa*. Moreover, multi-drug-resistant *S. globosa* strains are refractory to conventional antifungal therapy. The present study explores the anti-sporotrichotic effects of the essential oil of *Artemisia gmelinii* leaves (AgLEO) against a multi-drug-resistant strain of *S. globosa*.

**Methods:**

Gas chromatography–tandem mass spectrometry (GC-MS/MS) was performed for chemical profiling of AgLEO. Broth microdilution assay determined the inhibitory concentration of AgLEO against *S. globosa*. Oxidative stress in yeast was evaluated using the H2-DCFDA assay. Calcofluor white staining highlighted morphological changes in *S. globosa* under a fluorescent microscope. Ultra-high-performance liquid chromatography (UHPLC) determined total ergosterol levels in *S. globosa*. Biofilm formation was assessed by lactophenol cotton blue staining followed by brightfield microscopy.

**Results:**

GC-MS/MS analysis revealed that AgLEO is rich in oxygenated monoterpenes, sesquiterpene hydrocarbons, monoterpene hydrocarbons, and oxygenated sesquiterpenes. *S. globosa* yeast cells exhibited a dose-dependent susceptibility to AgLEO, with minimum inhibitory concentrations (MIC_50_ and MIC_90_) of 0.3% (v/v) and 0.4% (v/v), respectively. Mechanistic investigations demonstrated that AgLEO induces intracellular accumulation of reactive oxygen species (ROS) within 4 h of treatment, indicating oxidative stress-mediated cellular damages in *S. globosa*. Calcofluor white staining highlighted distorted and attenuated hyphal structures in *S. globosa* with AgLEO. Subsequently, UHPLC analysis also revealed a significant reduction of total ergosterol content in *S. globosa* with AgLEO. Together, these findings suggest that AgLEO-induced loss of structural integrity in *S. globosa* impaired the biofilm formation in *S. globosa* yeast cells.

**Conclusions:**

This study provides the first mechanistic evidence of AgLEO as an effective herbal agent against drug-resistant *S. globosa*. AgLEO exhibit multi-faceted anti-sporotrichotic effects by ROS induction, structural destabilization, ergosterol depletion, and biofilm inhibition. The outcome of the current study provides a promising botanical approach for sporotrichosis management and paves the way for developing plant-driven anti-sporotrichotic therapy.

## Graphical Abstract

**Figure:**
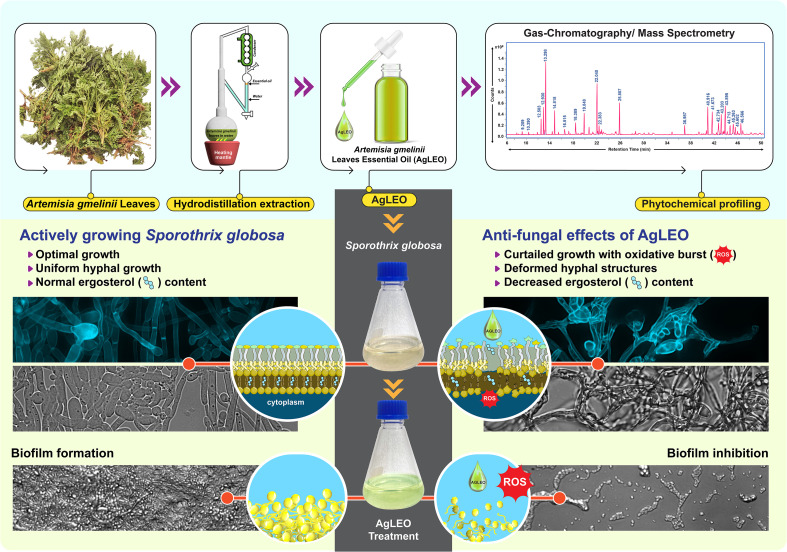
Proposed antifungal activity of AgLEO against *S. globosa*. AgLEO exerts antifungal activity through ROS generation, cellular and structural perturbations, ergosterol depletion, and inhibition of biofilm formation.

## 1 Introduction

Sporotrichosis is a subacute to chronic mycosis in both humans and domestic animals, such as cats and dogs, caused by different species of the fungal genus *Sporothrix*. Recognized by the World Health Organization (WHO) as a neglected tropical disease (NTD), sporotrichosis has been listed into the global programs addressing skin NTDs (Smith et al., 2025). Classically considered a sapronotic infection, sporotrichosis has long been referred to as “rose gardener’s disease” due to its transmission through soil, thorns, and decaying vegetation, commonly encountered during outdoor activities. Predominantly affecting individuals engaged in agriculture, horticulture, and veterinary care, it is also recognized as an occupational dermatosis (Foerster, 1926). Although consisting of approximately 53 species, the *Sporothrix schenckii* species complex forms pathogenic lineage of clinical relevance while other species are still thought to be associated with environmental clade (Rodrigues et al., 2020). The *S. schenckii* species complex mainly consists of cryptic pathogenic species, namely, *S. schenckii sensu stricto*, *S. brasiliensis*, *S. globosa*, *S. mexicana*, and *S. lurie*. Despite their morphological similarities, these species may exhibit notable differences in host preferences, virulence, pathogenicity, and drug resistance patterns. These interspecies variations emphasize upon the precise identification of *Sporothrix* at the species level under clinical and epidemiological settings. This is further substantiated with a peculiar global distribution pattern of the cryptic species due to their differential ecological preferences. *S. mexicana* has limited clinical reports in Portugal and Mexico. Likewise, *S. luriei*, although pathogenic, is exceedingly rare, with clinical cases reported from South Africa, Italy, and India (Fuchs et al., 2024). Notably, majority of sporotrichosis cases worldwide are attributed to three etiological agents, namely, *S. schenckii*, *S. brasiliensis*, and *S. globosa*. *S. brasiliensis* is historically considered endemic to Brazil, but recent evidence suggests its spread to proximal countries, such as Argentina, Paraguay, Uruguay, Chile, and Panama (Gómez-Gaviria et al., 2023), whereas *S. schenckii* is a cosmopolitan species, which exhibits a broad distribution spanning Australia, multiple European countries, South Africa, and most of the United States, consisting of the south, central and north regions of America (Rodrigues et al., 2020). *S. globosa* is another notable species with both zoonotic and sapronotic transmission routes, accounting for a substantial number of sporotrichosis cases reported across a wide geographic range distributed across Asia and parts of Europe and America. Prevalent in India, China, and Japan, *S. globosa* has also been reported in Spain, Guatemala, Mexico, Colombia, and Italy, highlighting this clinical clade as a globally emergent pathogen. Notably, *S. globosa* accounts for approximately 56% of sporotrichosis cases in Asia and exhibits significant endemicity in India, since all reported strains from India are linked to *S. globosa* (Nava-Pérez et al., 2022). Taken together, the widespread presence of *S. globosa*, coupled with the increasing resistance to conventional antifungal agents, makes sporotrichosis an emerging health concern worldwide. Cutaneous infections of sporotrichosis are typically chronic and treated with the first-line antifungal itraconazole; however, severe infections may even require intravenous Amphotericin B (Amp B) treatment along with surgical interventions. Given the treatment limitations and the rising burden of drug resistance, particularly in Indian clinical settings, *S. globosa* is the focal species that needs to be addressed. Moreover, *S. globosa* in particular has shown reduced susceptibility to several commonly used antifungal agents, including Amp B, fluconazole, voriconazole, and itraconazole. As a result, there is growing interest in developing an effective prophylactic or therapeutic vaccine for managing sporotrichosis (Nava-Pérez et al., 2022).

Several plant extracts and essential oils (EOs) have shown promising antifungal activity against *Sporothrix* species, particularly *S. schenckii*. A review by Waller et al. has listed 141 plant species from 39 botanical families with anti-*Sporothrix* activity. The largest number of these plant species belong to Combretaceae, Asteraceae, Lamiaceae, Fabaceae, Myrtaceae, Piperaceae, and Zygophyllaceae botanical families. These findings highlight plants as potential, stand-alone, or adjunct therapeutic options for the management of sporotrichosis, particularly in cases with resistance to conventional antifungal drugs (Waller et al., 2016). However, despite promising *in vitro* results, there are gaps in the methodologies, establishment of proof of concept, and mechanisms. Moreover, the emergence of drug-resistant *S. globosa* strains underlines the urgent need for such research, especially to explore plant-derived compounds as alternative or adjunct antifungal therapies.

Considering high clinical relevance and prevalence, particularly in India and other Asian regions, *S. globosa* was selected as a representative species of the *Sporothrix* genus to test the antifungal effects of EO extracted from the leaves of *Artemisia gmelinii* (AgLEO). The pathogenicity of thermally dimorphic *S. globosa*, like the sister clade species *S. brasiliensis* and *S. schenckii*, is attributed to their ability to switch from a mycelial form at environmental temperatures (25–28°C) to a yeast form at mammalian body temperature (36–37°C). *A. gmelinii* leaves’ EO was tested against both yeast and hyphae forms of *S. globosa*. *A. gmelinii*, commonly referred to as Russian Wormwood, is a perennial, drought-tolerant plant known for its low maintenance requirements. It has a long-standing history of medicinal use in traditional Korean and Chinese medicine. In India, it is locally known as *Nagdona* or *Ganga Tulsi* and is widely distributed across various regions. *A. gmelinii* possesses diverse medicinal properties, including notable antibacterial, antifungal, anti-inflammatory, hepatoprotective, and antioxidant activities. *A. gmelinii* extract has also been studied in allergic airway inflammation in an OVA-induced combined allergic rhinitis and asthma syndrome (CARAS) mouse model (Nguyen et al., 2022). The ethanolic and chloroform extracts prepared using aerial parts of *A. gmelinii* demonstrated broad-spectrum efficacy against Gram-positive bacteria and fungus, *Candida* spp (Mamatova et al., 2019). EO from other species of *Artemisia*, namely, *A. stricta*, has been previously reported to impart antifungal effects against *Aspergillus flavus*, *Aspergillus niger*, and *S. schenckii* (Pandey and Singh, 2017). However, the study was preliminary. The current study has focused on highlighting the direct evidence supported by mechanistic aspects of EO from *A. gmelinii* leaves against drug-resistant *S. globosa*. The study outcomes would be instrumental in addressing the need to target drug resistance in *Sporothrix* spp.

## 2 Materials and methods

### 2.1 Fungal strain and culture conditions

The pathogenic strain of *S. globosa* (NCCPF220119) was procured from the National Culture Collection of Pathogenic Fungi (NCCPF), Post Graduate Institute of Medical Education & Research (PGIMER), Chandigarh, India. Fungal cells were reactivated from glycerol stock, first by streaking on brain heart infusion (BHI) agar plates for single colony and, subsequently, by overnight culturing in BHI broth at 37 °C for yeast form and at 25°C for filamentous form. All *S. globosa* cultures were grown either on BHI agar plates or in BHI broth. All culture media were procured from Himedia, India.

### 2.2 Plant procurement and extraction of essential oil from leaves of *Artemisia gmelinii*

*A. gmelinii* Weber ex Stechm. was collected from Chirwasa, Gangotri-Gaumukh, Uttarkashi, Uttarakhand, India. The plant was authenticated and verified by an experienced taxonomist at Patanjali Research Foundation Herbarium (PRFH) and recorded under the Collection# 5983 (PORI). The authenticated plant materials were washed with distilled water, air-dried, and subjected to an EO extraction process using hydro-distillation with a clevenger apparatus. Leaves weighing 1 kg were transferred to an extraction flask followed by the addition of 10 L of distilled water. After 1 to 2 h of soaking, heat (95–100 °C) was applied for nearly 4–5 h to induce vaporization and subsequent condensation. The latter was passed through separating funnel to collect EO in a collection vessel. The obtained EO was labeled with in-house batch number PRF/CHI/0424/0288 (abbreviated as AgLEO) and stored at stored at 4 °C. The percentage yield of EO obtained from the process was calculated as below:

[(Essential oil volume (mL))/(Sample weight (g))] × 100.

### 2.3 Determination of minimum inhibitory concentration

The antifungal effect of AgLEO against *S. globosa* was determined through the broth microdilution method as descried previously (Balkrishna et al., 2025). AgLEO at 4% (v/v) was suspended in BHI media. A subsequent dilution at 2.0% was also prepared in culture media, which was then twofold diluted serially in a 96-well plate. To 100 µL of AgLEO dilution, each well then received another 100 µL of either BHI broth alone (media blank control) or *S. globosa* inoculum prepared in BHI broth to a final yeast cell density of 1 × 10^6^ yeast cells/mL. Wells containing *S. globosa* inoculum but no AgLEO served as the untreated positive control. After incubation at 37°C for 24 h, absorbance at 600 nm was measured using a microplate reader (Envision, Perkin Elmer, USA). The percentage of growth inhibition in *S. globosa* with AgLEO treatment was determined with respect to the untreated one. To eliminate any background interference, all readings were corrected by subtracting their respective blank values (blank correction). Datasets represent mean ± SEM from three independent replicates (*n* = 3), each with three technical replicates. Further analyses were performed in Prism 8.0.2 software (GraphPad Software Inc., USA). Minimum inhibitory concentration (MIC) of AgLEO inhibiting 50% (MIC_50_) and 90% (MIC_90_) of *S. globosa* growth were determined by plotting non-linear regression curve fit.

### 2.4 Biofilm inhibition assay

Actively growing S*. globosa* inoculum was adjusted to 0.1 at OD_600_. For the biofilm inhibition assay, the culture was inoculated in 12-well plates containing sterile coverslips with or without AgLEO at 0.5×, 1.0×, and 2.0× MIC_50_. The plates were statically incubated for 72 h at 37 °C. Post-incubation, non-adherent yeast cells were removed by rinsing each well with 500 µL of sterile phosphate-buffered saline (PBS). Biofilms were stained with lactophenol cotton blue and images were captured under brightfield microscopy (400×, Eclipse Ts2, Nikon, Japan). The area covered by the biofilm was determined using ImageJ software.

The metabolic activity of the biofilms formed by *S. globosa* was evaluated using colorimetric Alamar blue assay. Biofilms were formed as described above. After 72 h of incubation, biofilms formed by *S. globosa* were thoroughly washed with 500 µL of PBS and incubated at 37 °C for 2 h with Alamar blue reagent (Himedia, India) at a final concentration of 50 µg/mL, prepared in the BHI broth. Reduction of resazurin in Alamar blue assay by metabolically active biofilms turns it pink, which can be quantitatively captured via fluorescence intensity at an excitation/emission of 560/590 nm (Infinite 2000 Pro microplate reader, Tecan Group Ltd., Switzerland). The obtained arbitrary fluorescent units were expressed as a percentage change in metabolic activity compared to the untreated one (positive control). All obtained fluorescent values were blank corrected.

### 2.5 Ultra-high performance liquid chromatography coupled with diode array detection-based quantification of total ergosterol content in *S. globosa*

*S. globosa* cells were treated with AgLEO at different concentrations, 0.07% (v/v), 0.15% (v/v), 0.3% (v/v), and 0.6% (v/v), corresponding to 0.25×, 0.5×, 1.0×, and 2.0× MIC_50_ of AgLEO, respectively. After 24 h of treatment, *S. globosa* cells were centrifuged at 4,000 rpm for 5 min in 15-mL tubes. The obtained yeast pellet was then washed twice with 5.0 mL of autoclaved Milli-Q water. Each pellet was independently weighed and resuspended in 500 µL of methanol. Steel beads were added and cells were homogenized in a tissue lyser for 15 min. Lysed cells were again centrifuged at 10,000 rpm for 10 min to remove the debris; supernatant collected was subjected to ultra-high-performance liquid chromatography (UHPLC) coupled with diode array detection (UHPLC-DAD) for ergosterol quantification against standard. Ergosterol standard (TCI, India) of 1,000 parts per million (ppm) was prepared in methanol. 10 ppm working standard solution was made by diluting 1,000 ppm stock solution. The quantification of ergosterol was performed by UHPLC (Prominence-i LC-2030c 3D Plus, Shimadzu, Japan). Separation was achieved using a VDSpher PUR 120 C18-U (5 µm, 4.6 × 250 mm) column subjected to isocratic elution with a flow rate of 1.0 mL/min. The mobile phase was used for the analysis of methanol:acetonitrile ratio (80:20). Standard and test solution (50 µL) was injected and wavelength was set at 280 nm. Absolute ergosterol quantification in *S. globosa* was normalized to the pellet weight and expressed as µg/mg of pellet weight. Datasets represent mean ± SEM from three independent experiments.

### 2.6 Detection of intracellular reactive oxygen species

ROS generation in *S. globosa* was evaluated using the 2′,7′-dichlorofluorescin diacetate (DCFDA) assay. Briefly, actively growing yeast cells corresponding to 0.1 OD_600_ were treated with AgLEO at different concentrations [0.07% (v/v), 0.15% (v/v), 0.3% (v/v), and 0.6% (v/v), corresponding to 0.25×, 0.5×, 1.0×, and 2.0× MIC_50_ of AgLEO, respectively], antifungal control Amp B (at 0.5 µg/mL), positive control for oxidative burst, and Vitamin K3 (Menadione, VK3) at 100 µM for 4 h at 37 °C. After 3.5 h, 50 µM DCFDA (H2-DCF-DA; D6883, Sigma-Aldrich, USA) was added to all treatments and kept for 30 min at 37 °C. After incubation, yeast cells were centrifuged at 4000 rpm for 5 min and resuspended in PBS (pH 7.4). ROS levels were quantified by measuring fluorescence intensity using a microplate reader (Envision, Perkin Elmer, USA) at excitation/emission wavelengths of 485/535 nm. Fold change in ROS levels was calculated with respect to the untreated cells that served as negative controls.

### 2.7 Calcofluor white staining of *S. globosa*

*S. globosa* at 1×10^6^ cells/mL were treated at different concentrations of AgLEO (0.6% v/v, 0.3% v/v, and 0.15% v/v). After 24 h of treatment at 25°C, *S. globosa* cultures were collected in 1.5-mL tubes and centrifuged at 4000 rpm for 5 min. Following a rinse with 1.0 mL of PBS, pelleted *S. globosa* culture was fixed with 2.0% formaldehyde for 15 min at room temperature before staining with Calcofluor white (CFW) stain (Sigma-Aldrich, 25 μg/mL) for 10 min in the dark. After staining, cells were washed once with 1.0 mL of PBS to remove excess dye and *S. globosa* culture was mounted on glass slides. Fluorescence was observed under a fluorescence microscope [APEXVIEW APX100 benchtop fluorescence microscope, Evident Scientific (previously Olympus), USA] using a DAPI filter set with excitation/emission of ~355 nm/~433 nm to visualize chitin-rich cell walls at 40× and 100× magnification.

### 2.8 Mammalian cell line maintenance and viability assay

Human keratinocytes and HaCaT cells were procured from Krishgen Biosystems, India (Cat # KCC0090). The cell lines were routinely maintained in phenol-red-free DMEM (Dulbecco’s Modified Eagle Medium) containing 10% fetal bovine serum (FBS) and 1% Anti–Anti (Antibiotic–Antimycotic) under growing conditions maintained at 37 °C and 5% CO_2_ in a humified incubator. Cryopreserved cells were passaged at least twice before use for the experiments in this study. Cells (1 × 10^5^ cells/mL) were seeded in 96-well plates and incubated for 24 h. Cells were then treated with AgLEO ([0.01–1% (v/v)]; half-log dilutions) for 24 h. Alamar Blue at a final concentration of 15 μg/mL was added 3 h before the endpoint. Fluorescence was measured at Ex/Em 560/590 nm using an EnVision multi-plate reader (PerkinElmer, USA). Percent cell viability was calculated after blank correction using untreated cells as control (Balkrishna et al., 2024).

### 2.9 Gas chromatography–mass spectrometry-based analysis of AgLEO

To identify constituents present in AgLEO, the latter was subjected to GC-MS/MS-based analysis (7000D GC/MS triple quad with 7890B GC system, Agilent, USA with mass hunter software). AgLEO (50.1 mg) was transferred to a 2.0-mL glass vial, followed by the addition of 2.0 mL of ethyl acetate. The mixture was thoroughly mixed and sonicated for 5 min in ice-cold water in a water bath sonicator (Fisher Scientific, USA) at a frequency of 37 kHz. Separation was performed using an Agilent HP-5 MS capillary column (30 m × 0.25 mm, 0.25 µm). The carrier gas, helium, passed at a flow rate of 1 mL/min. The temperature of the split injector was maintained at 280°C with a split ratio of 20:1. The injection volume was 1 μL. The column temperature was set at 60 °C (held for 3 min) then changed at 2 °C/min to 150 °C (held for 5 min) and followed by 6 °C/min to 210 °C (held for 2 min). The GC-MS ion source temperatures and ionization potential was maintained at 230 °C and 70 eV, respectively.

### 2.10 Statistical analysis

The statistical analysis was performed on Microsoft^®^ Excel^®^ (Version 2507 Build 16.0.19029.20136) and GraphPad Prism (GraphPad Software, version 8.0.2, USA). Datasets were presented as the mean ± standard error of mean (SEM) from at least three biological replicates each with technical triplicates. Multi-group comparisons were conducted using one-way analysis of variance (ANOVA) followed by Dunnett’s multiple comparisons test. The results were deemed significant when *p* < 0.05.

## 3 Results

### 3.1 Compositional analysis of AgLEO using gas chromatography–tandem mass spectrometry

**Figure 1:**
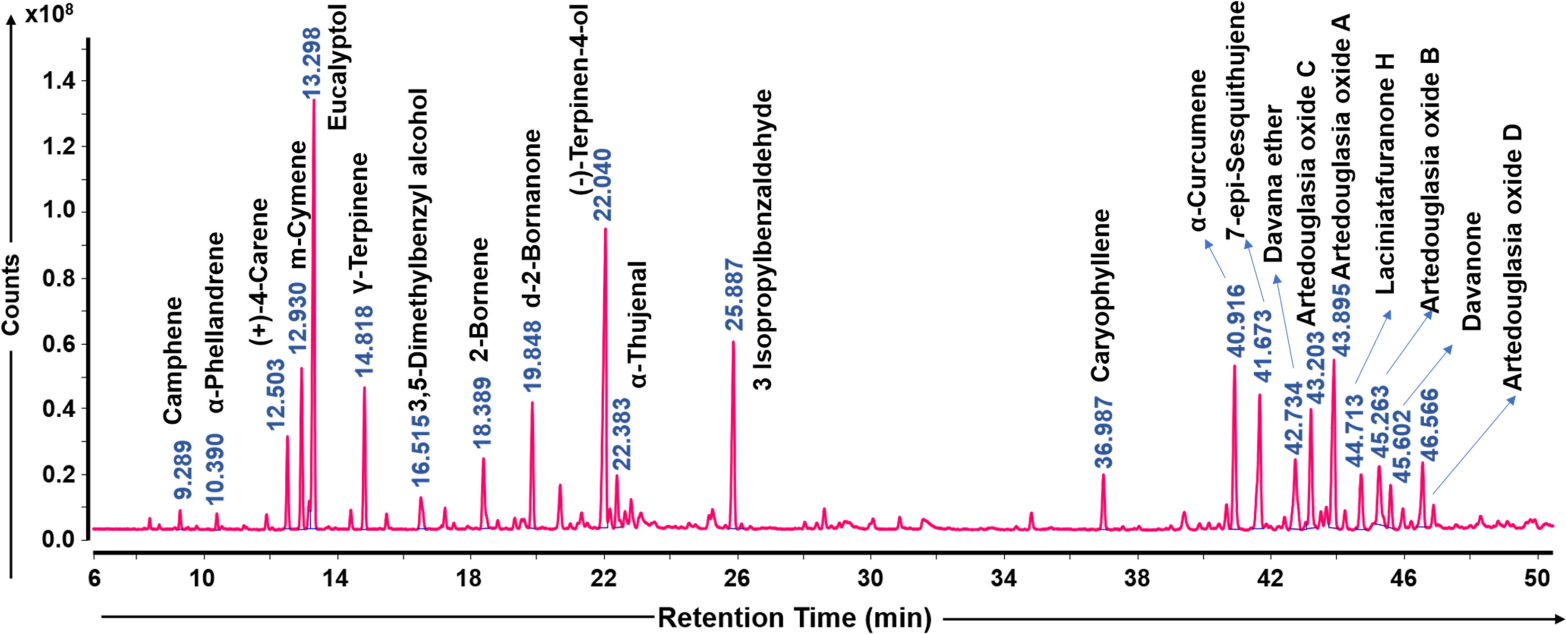
Gas chromatography–tandem mass spectrometry (GC-MS/MS) analysis of essential oil extracted from *Artemisia gmelinii* leaves (AgLEO). Representative chromatograms (pink lines) of AgLEO, illustrating the retention times of identified phytometabolites (in blue text). Peaks corresponding to phytocompounds have been annotated in black text, with detailed tabulation in **Table 1**.

The EO yield using the hydro-distillation method from leaves of *A. gmelinii* was 0.42% (v/w). GC-MS/MS analysis was conducted to determine the overall phytochemical composition of EO from leaves of *A. gmelinii* (AgLEO). The GC-MS/MS chromatogram shown in **Figure 1** and the corresponding phytometabolite list tabulated in **Table 1** revealed 22 volatile constituents, predominantly non-polar to moderately polar compounds, collectively representing the chemical profile of AgLEO. The phytometabolites identified in the chromatogram were confirmed using retention times and mass spectra comparison with the Standard Reference Data Program of the National Institute of Standards and Technology (NIST) Mass Spectral Library, version 2.2 (Gaithersburg, USA). The relative abundance of each component was calculated as the percent area of total ion chromatogram peaks. Based on the chemical structures of the identified compounds, oxygenated monoterpene comprised the largest fraction in AgLEO, followed by monoterpene hydrocarbons and then sesquiterpenes, which is consistent with the distribution patterns typically observed in EOs (El-Nashar et al., 2021). The collective content of oxygenated monoterpenes was identified in a substantial amount of 41.05% largely consisting of eucalyptol (13.92%), d-2-bornanone (4.28%), (–)-terpinen-4-ol (13.92%), α-thujenal (1.87%), and 3-isopropylbenzaldehyde (7.06%), whereas monoterpene hydrocarbons were 15.44% in abundance, composed of camphene (0.46%), α-phellandrene (0.39%), (+)-4-carene (2.64%), m-cymene (4.79%), γ-terpinene (4.26%), and 2-bornene (2.90%). Sesquiterpene hydrocarbons consisted of caryophyllene (2.10%), α-curcumene (6.44%), and 7-epi-sesquithujene (6.39%). Oxygenated sesquiterpenes consisted of davana ether (4.04%), artedouglasia oxides (18.97%), laciniatafuranone H (2.47%), and davanone (1.60%). The parenthesis indicates the relative abundance of each component as percent peak area of the total ion chromatogram.

**Table 1:** List of phytometabolites identified in essential oil from *Artemisia gmelinii* leaves (AgLEO) on the GC-MS/MS platform.

S. no.	Name of the phytometabolite	RT	Formula	Relative area (%)
1	Camphene	9.289	C_10_H_16_	0.46
2	α-Phellandrene	10.39	C_10_H_16_	0.39
3	(+)-4-Carene	12.503	C_10_H_16_	2.64
4	m-Cymene	12.93	C_10_H_14_	4.79
5	Eucalyptol	13.298	C_10_H_18_O	13.92
6	γ-Terpinene	14.818	C_10_H_16_	4.26
7	3,5-Dimethylbenzyl alcohol	16.515	C_9_H_12_O	1.51
8	2-Bornene	18.389	C_10_H_16_	2.90
9	d-2-Bornanone	19.848	C_10_H_16_O	4.28
10	(−)-Terpinen-4-ol	22.04	C_10_H_18_O	13.92
11	α-Thujenal	22.383	C_10_H_14_O	1.87
12	3-Isopropylbenzaldehyde	25.887	C_10_H_12_O	7.06
13	Caryophyllene	36.987	C_15_H_24_	2.10
14	α-Curcumene	40.916	C_15_H_22_	6.44
15	7-epi-Sesquithujene	41.673	C_15_H_24_	6.39
16	Davana ether	42.734	C_15_H_22_O_2_	4.04
17	Artedouglasia oxide C	43.203	C_15_H_22_O_3_	4.90
18	Artedouglasia oxide A	43.895	C_15_H_22_O_3_	7.84
19	Laciniatafuranone H	44.713	C_15_H_22_O_3_	2.47
20	Artedouglasia oxide B	45.263	C_15_H_22_O_3_	3.38
21	Davanone	45.602	C_15_H_24_O_2_	1.60
22	Artedouglasia oxide D	46.566	C_15_H_22_O_3_	2.85

The respective chromatogram has been depicted in **Figure 1**.

### 3.2 AgLEO impedes *S. globosa* growth by upregulating intracellular ROS

**Figure 2:**
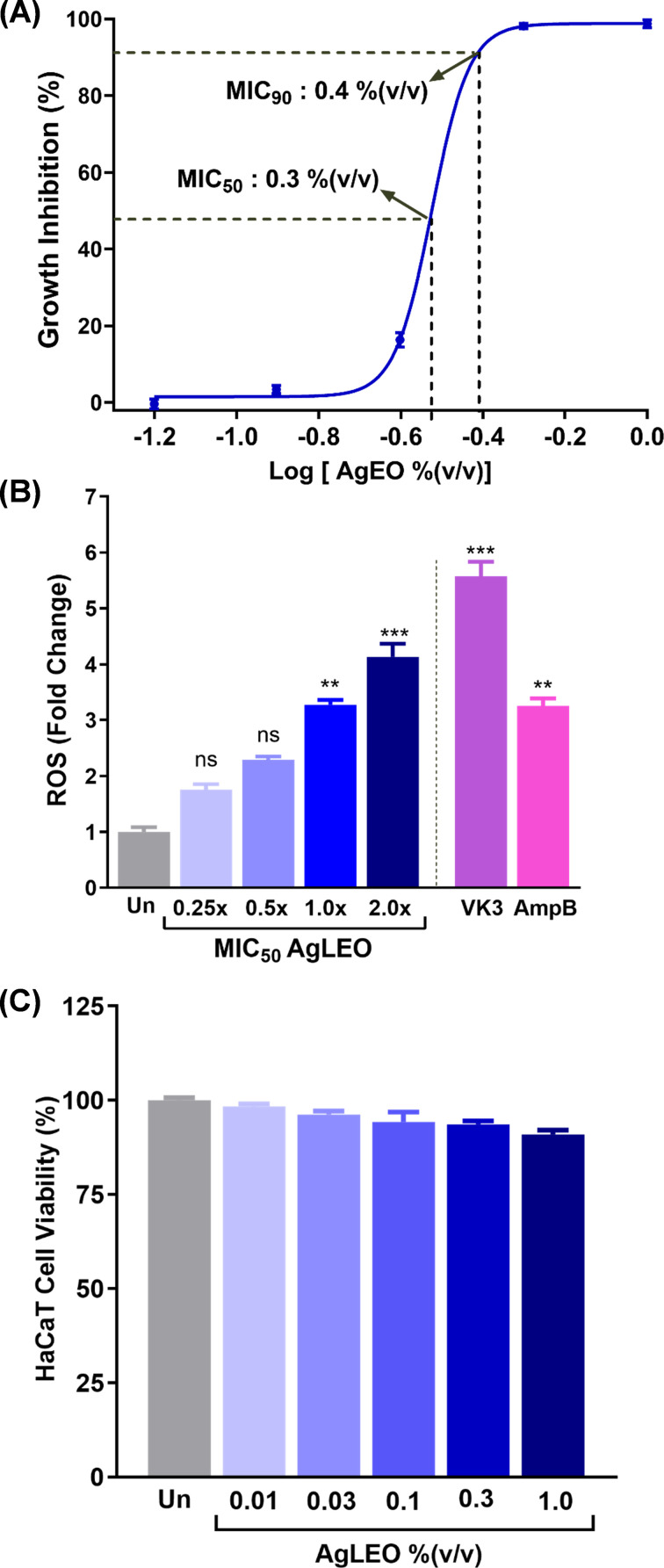
Antifungal effects of AgLEO against *S*. *globosa*. **(A)** Dose–response curve indicating MIC_50_ and MIC_90_ for AgLEO (indicated with arrows) against *S. globosa*. Datasets from three independent experiments represent mean ± SEM. **(B)** Bar graph representing intracellular ROS levels in *S. globosa* yeast cells treated with AgLEO at 0.25× [0.07% (v/v)], 0.5× [0.15% (v/v)], 1.0× [0.3% (v/v)], and 2.0× [0.6% (v/v)] MIC_50_. Un: Untreated. Error bars represent mean ± SEM from three independent experiments; significance of data has been represented as **p < 0.01, ***p < 0.001, and ns for non-significant. **(C)** Biocompatibility of AgLEO in HaCaT cells. Data are presented as mean ± SEM from three independent experiments. Un: Untreated.

To address the antifungal effect of AgLEO against the pathogenic yeast form of *S*. *globosa*, broth microdilution assay was performed. Previously, our group has demonstrated that the *S*. *globosa* strain in the yeast form showed resistance against the antifungal drug itraconazole (Balkrishna et al., 2022). AgLEO supressed 50% (MIC_50_) and 90% (MIC_90_) growth of the same *S. globosa* strain at a concentration of 0.3% (v/v) 0.4% (v/v), respectively (**Figure 2A**). Following the determination of the MIC_50_ and MIC_90_ values, to elucidate the mechanism underlying AgLEO-mediated growth inhibition, ROS generation in *S. globosa* yeast cells was evaluated. Amp B has been previously evaluated to exhibit MIC_50_ at 0.55 μg/mL in the *S. globosa* strain used in this study. *S. globosa* yeast cells were treated with Amp B at 1.0× MIC_50_, and AgLEO at varying concentrations of 0.25× [0.07% (v/v)], 0.5× [0.15% (v/v)], 1.0× [0.3% (v/v)], and 2.0× [0.6% (v/v)] of MIC_50_ for 4 h. Menadione (VK3) at 100 μM was included as a positive control. AgLEO dose-dependently and significantly increased the intracellular ROS levels up to three- to fourfold at 1.0× MIC_50_ (*p* < 0.01) and 2.0× MIC_50_ (*p* < 0.001) concentration of AgLEO (**Figure 2B**). Notably, the antifungal drug Amp B also elicited a nearly threefold (*p* < 0.01) increase of ROS in *S. globosa* (**Figure 2B**). Subsequently, human skin keratinocytes (HaCaT cells) were exposed to AgLEO to assess its biocompatibility. AgLEO at the highest tested concentrations [1.0% (v/v)] showed no significant effects on HaCaT cell viability, which was comparable with the untreated cells, suggesting AgLEO’s biocompatibility with the mammalian cells at the active antimicrobial range with MIC_90_ at 0.6% (v/v) (**Figure 2C**). Collectively, the data indicate oxidative stress as a potential mediator of the inhibitory effect of AgLEO and Amp B in *S. globosa*.

### 3.3 AgLEO disrupts the structural integrity in *S. globosa*

**Figure 3:**
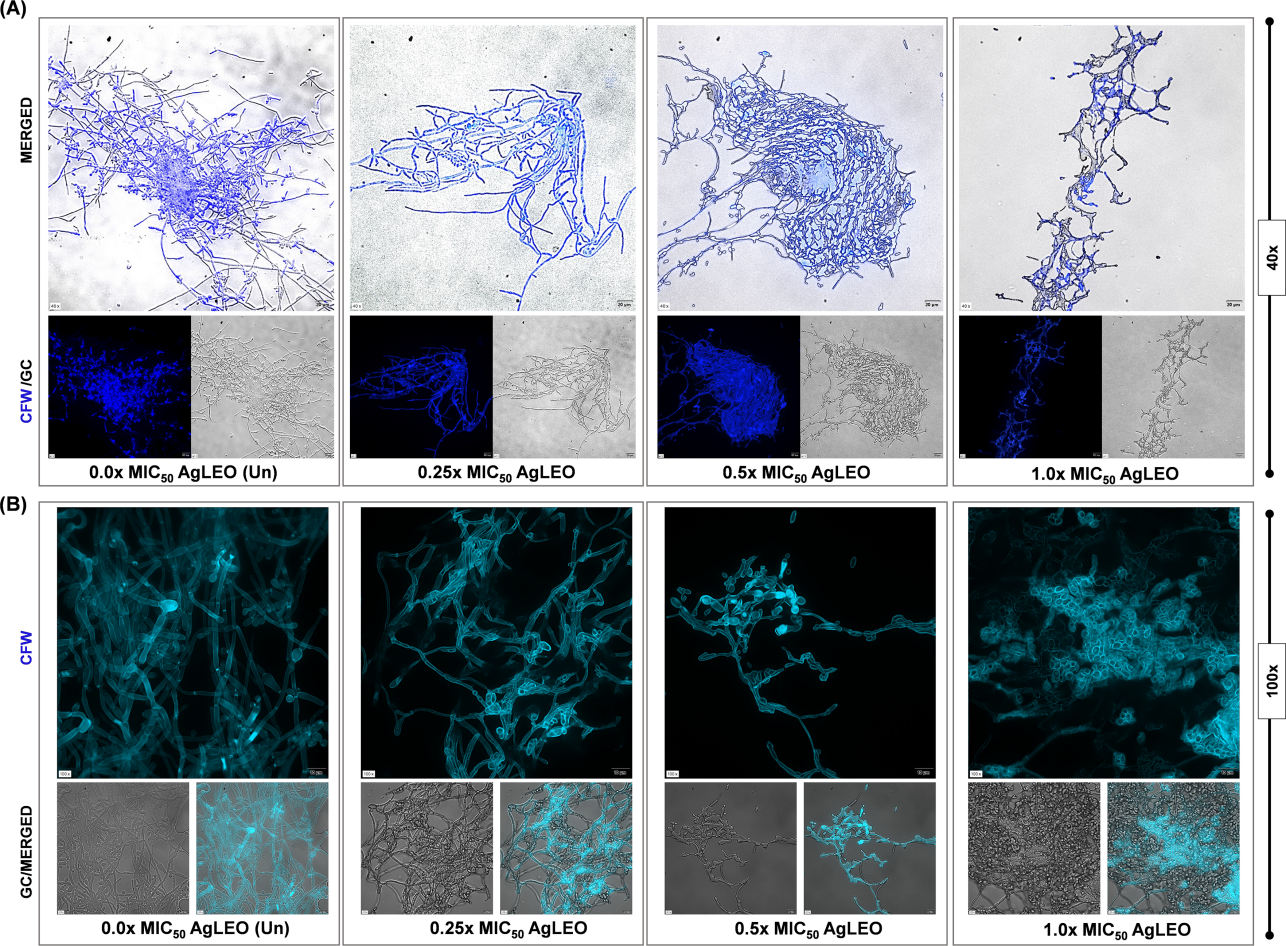
Calcofluor white (CFW) staining of *S. globosa*. Images captured at 40× **(A)** and 100× **(B)** magnification. Images of CFW staining (blue/cyan) were captured using the DAPI filter. The corresponding brightfield images [gradient contrast (GC)] were also captured to visualize the overall hyphal growth.

CFW is a fluorescent dye that binds to β-1,3 and β-1,4 polysaccharides in chitin and cellulose present in the fungal cell walls to enable visualization of morphological features and integrity under a fluorescence microscope (Rasconi et al., 2009). Yeast cells and fungal hyphae of *S. globosa* were treated with 0.25× MIC_50_, 0.5× MIC_50_, and 1.0× MIC_50_ of AgLEO and visualized at 40× and 100× magnification under a fluorescent microscope (**Figure 3**). Imaging at 40× magnification provided a comparative overview of hyphal network and general morphology of *S. globosa* in the presence of AgLEO. Marked morphological alterations in *S. globosa* hyphae, including disrupted, irregular, deformed, and shrunk structures, were observed in *S. globosa* when treated with AgLEO while uniform filamentous growth was observed in the untreated *S. globosa* (**Figure 3A**). At 100× magnification, detailed visualization revealed pronounced structural damage in *Sporothrix* cells following AgLEO treatment. Irregular hyphal contours, collapsed, clumped cell walls, and surface deformities were clearly visualized upon AgLEO treatment not only under fluorescent but also with the gray contrast. Notably, the uniformity of CFW was lost with AgLEO treatment, clearly indicating structural distortions, loss of structural integrity, and disruption in chitin and cellulose levels where CFW binds (**Figure 3B**).

### 3.4 AgLEO substantially alters ergosterol contents in *S. globosa*

**Figure 4:**
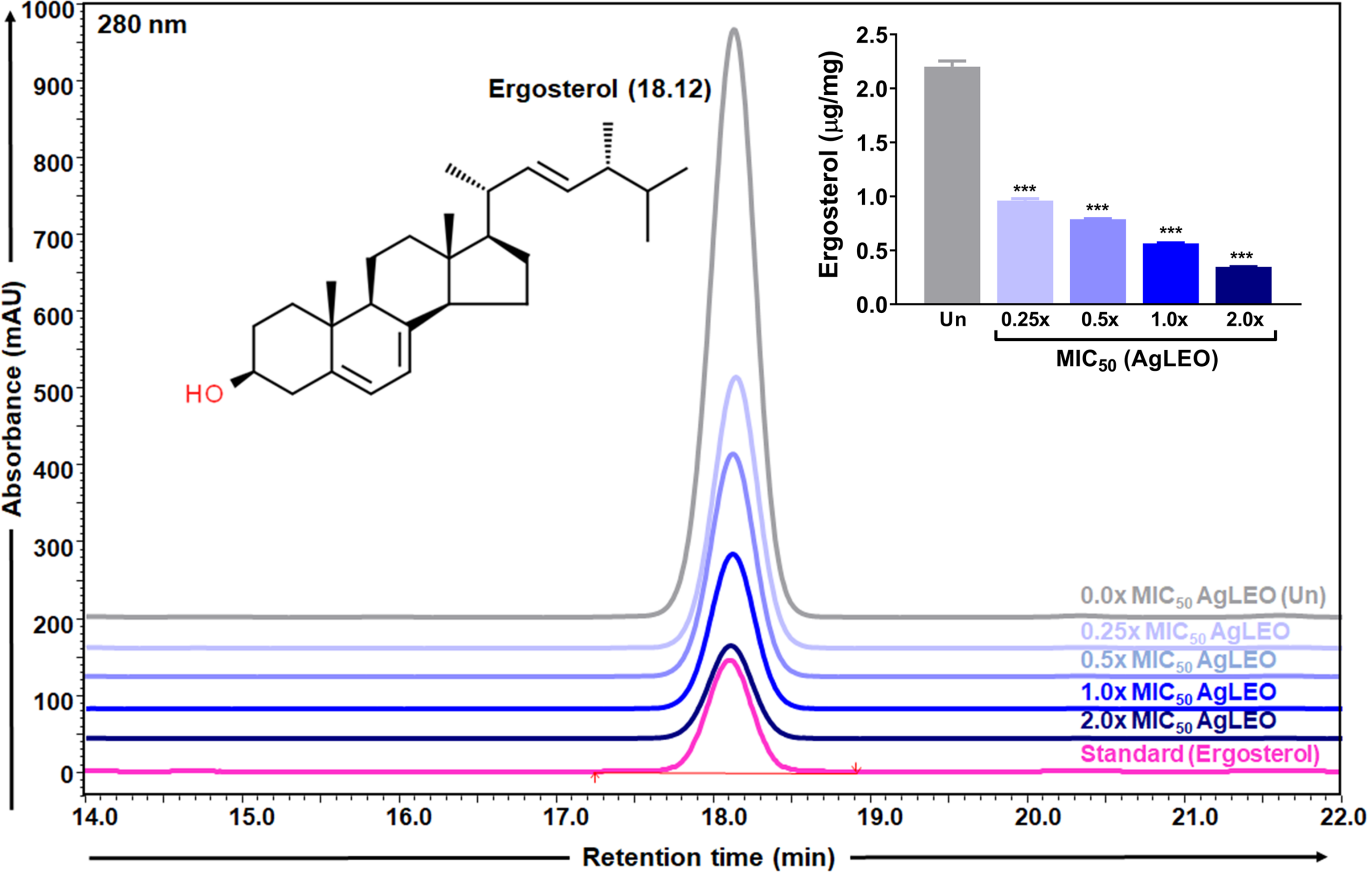
Total ergosterol content in *S*. *globosa*. Representative chromatogram for ergosterol detection was recorded at 280 nm using UHPLC-DAD. The chromatograms displaying peaks corresponding to ergosterol content detected in *S. globosa*, treated with indicated concentrations of AgLEO. Quantification performed with reference to the ergosterol standard (pink). Bar graph (top right) provides quantification of ergosterol content, normalized with *S. globosa* pellet weight (in mg). Error bars represent mean ± SEM from three independent experiments; significance of data has been represented as ***p < 0.001.

Ergosterol is one of the principal sterols present in fungal cell membrane. Ergosterol imparts structural integrity and fluidity to fungal cell membranes and plays a critical role in maintaining membrane permeability and several critical physiological functions (Parks et al., 1995; Dupont et al., 2012; Eliaš et al., 2024). Altered ergosterol levels imbalance the cellular homeostasis impeding the fungal growth but enhancing their vulnerability toward exogenous stress conditions (Parks et al., 1995). AgLEO-mediated alterations in ergosterol levels were quantitatively evaluated using UHPLC-DAD. At 0.25× MIC_50_ AgLEO, *S. globosa* lost half of the total ergosterol levels compared to untreated cells (**Figure 4**). A significant (*p* < 0.001) dose-dependent reduction in the total ergosterol levels at 0.25× MIC_50_, 0.5× MIC_50_, 1.0× MIC_50_, and 2.0× MIC_50_ of AgLEO corroborates the loss of structural integrity in *S. globosa* with AgLEO.

### 3.5 Antibiofilm activity of AgLEO against *S. globosa*

**Figure 5:**
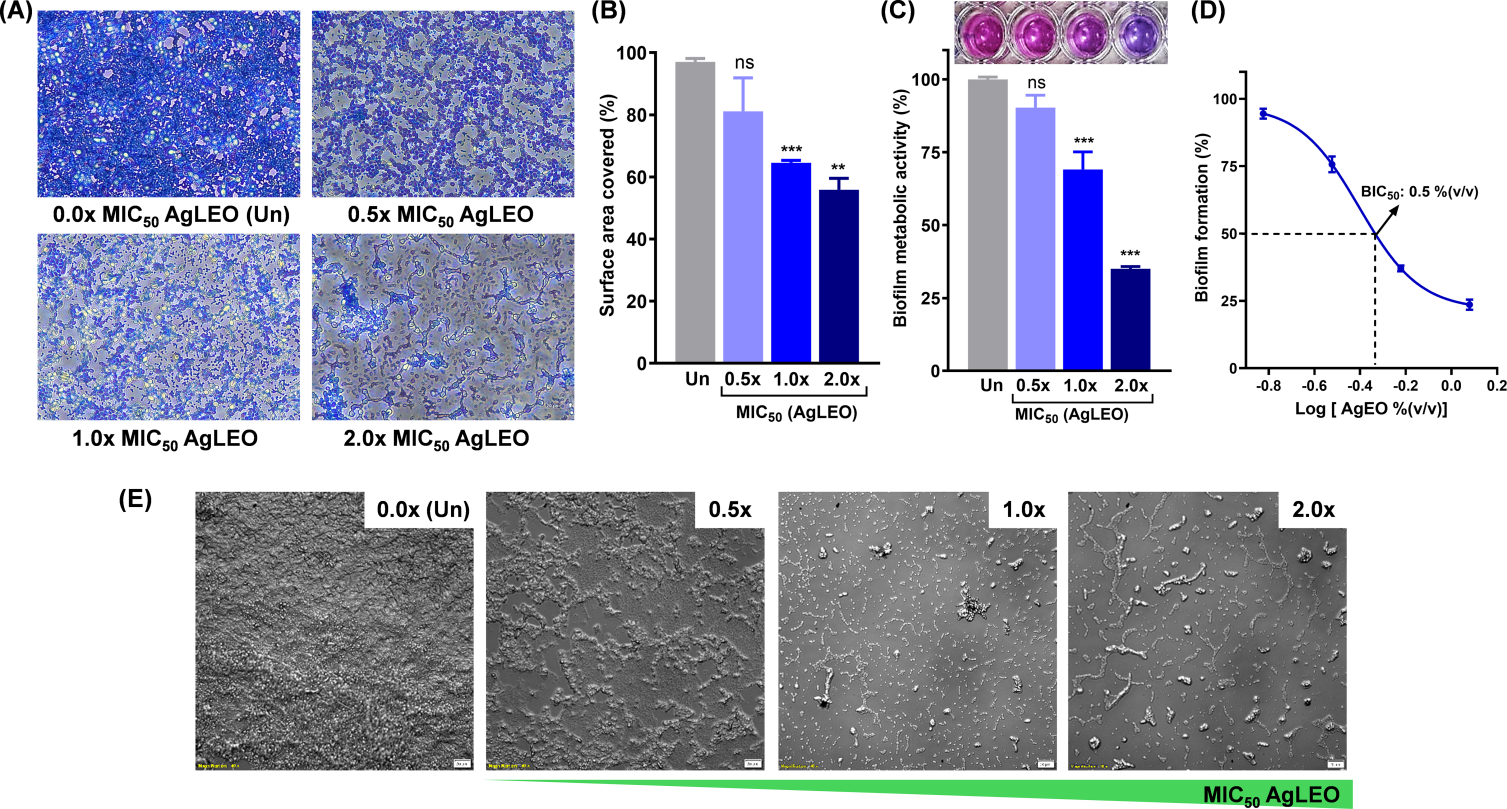
AgLEO inhibits biofilm formation of *S. globosa*. **(A)** Yeast *S. globosa* cells treated with AgLEO at increasing concentrations (0.25×, 0.5×, 1.0×, and 2.0× MIC_50_). Representative brightfield microscopy images show biofilms formed by untreated (Un) and AgLEO-treated yeast cells, after lactophenol blue staining. Untreated (Un) *S. globosa* biofilm shows dense coverage, while AgLEO treatment (0.5×, 1.0×, and 2.0× MIC_50_) progressively induced disruption of biofilm. Scale bar: 20 µm **(B)** Bar graph depicts surface area coverage of *S. globosa* biofilms measured using the ImageJ software suite. **(C)** Alamar blue assay-based metabolic activity of biofilms formed by *S. globosa* in the presence of AgLEO. Representative images depicting the resazurin-to-resorufin color conversion in biofilms have also been represented on the top of the graph. **(D)** Non-linear regression curve fit indicating the biofilm inhibitory concentration (BIC_50_) of AgLEO. Error bars represent mean ± SEM from three independent experiments; significance of data compared with untreated (Un), represented as **p < 0.01, ***p < 0.001 and ns: non-significant. **(E)** Brightfield images [gradient contrast (GC)] at 40× magnification illustrate the biofilm structure and surface coverage formed by *S. globosa*.

It is well established that biofilms enhance drug resistance and escape from host immune defense mechanisms (Liu et al., 2024). The potential of yeast cells to develop biofilms contributes to persistent infection and treatment challenges. AgLEO targets *S. globosa* structural integrity; therefore, we hypothesized its inhibitory effect on the biofilm formation capacity of *S. globosa*. Lactophenol cotton blue stain binds specifically to chitin in the fungal cell walls, thereby imparting a blue color to yeast cells and biofilm structures. AgLEO treatment disorganized and interfered with the biofilm formation by the pathogenic yeast form of *S. globosa* (**Figure 5A**). Biofilm surface area in untreated *S. globosa* (97.1%) reduced to 81.1% (ns, non-significant), 64.6% (*p* < 0.001), and 55.9% (*p* < 0.01) when treated with 0.5× MIC_50_, 1.0× MIC_50_, and 2.0× MIC_50_ of AgLEO, respectively (**Figure 5B**). The metabolic activity of the biofilm was further assessed using Alamar blue assay, wherein viable and active biofilms show pink color development, whereas reduced viability show blue coloration. Compared to the untreated *S. globosa* biofilms (94.5%), the metabolic activity showed a substantial reduction to 37.0% (*p* < 0.001) and 23.6% (*p* < 0.001) at 0.5× MIC_50_, 1.0× MIC_50_, and 2.0× MIC_50_ of AgLEO treatment, respectively (**Figure 5C**). Wells showing Alamar blue color change with respect to viability and metabolic activity in *S. globosa* biofilms (**Figure 5C**; image below graph). Using the obtained metabolic activity, a non-linear regression curve fit was generated to calculate the biofilm inhibitory concertation (BIC_50_) of AgLEO that inhibits 50% of the biofilm metabolic activity. The curve showed BIC_50_ at 0.5% (v/v), which is higher than MIC_50_ [0.3% (v/v)] (**Figure 5D**). The biofilms were also imaged under a brightfield microscope in gray contrast to highlight the spatial organization of biofilm formation by *S. globosa*. AgLEO-mediated dose-dependent disruption in biofilm structures with sparse and irregular yeast cell arrangement was observed compared to the dense and well-organized biofilm formed in untreated control yeast cells (**Figure 5E**). The AgLEO-treated biofilms appeared thinner, with evident gaps with loss of interconnections and weakened structural integrity. These morphological changes demonstrate AgLEO with potent antibiofilm activity against *S. globosa*.

## 4 Discussion

Members of the genus *Artemisia* include a wide spectrum of species that are distributed across the globe except Antarctica. Rich with a myriad of bioactive compounds, *Artemisia* species have long been utilized and traditionally employed for its antimalarial, antioxidant, anticancer, antinociceptive, anti-inflammatory, and antiviral properties (Sharifi-Rad et al., 2022). *A. gmelinii* Webb & Stechmann, also known as Gmelin’s wormwood, is one of more than 500 species belonging to the genus *Artemisia* (family Asteraceae) (Mamatova et al., 2019). The leaves and stem of *A. gmelinii* are lush, mossy, and cushiony, which are traditionally used for the prevention and treatment of acute and chronic liver inflammation, hyperlipidemia, and infected cholecystitis. Previous research studies have also explored the antibacterial and antifungal potency of *A. gmelinii* (Yuan et al., 2010; Mamatova et al., 2019). EOs are historically known for their usage in traditional medicines, cosmetics, culinary, and aromatic applications. Several studies worldwide have reported the chemical composition of EOs extracted from various *Artemisia* genera. However, the phytometabolic profile varies with part of the plant, cultivation period, developmental stage of the plant, provenance, and extraction method. With particular reference to EO extracted from *A. gmelinii*, previously published studies have demonstrated considerable variability in chemical profiles; however, caryophyllene consistently emerges as a recurrent constituent across studies (**Figure 1**). Other major phytometabolites identified include γ-amorphene, isohumbertiol B, caryophyllene oxide, caryophylla-4 (Balkrishna et al., 2024), 8 (El-Nashar et al., 2021)-diene-5α-ol, ylangenol, cabrevia oxide B, cyclobutaneethanol, endo-borneol, germacrene D, eucalyptol, selin-6-en-4α-ol, bisabolone oxide A, terpinen-4-ol, isoascaridol, α-terpinolene, phellandrene, and ascaridole, reflecting both the chemical diversity and geographical or methodological influences on EO composition (Qadir et al., 2020; Xu et al., 2021; Zhigzhitzhapova et al., 2021). Notably, a study from Qadir et al. evaluated the phytochemical profile of aromatic constituents of *A. gmelinii* EO using GC-FID, GC-MS, and ^13^C NMR and reported the predominance of oxygenated monoterpenes (Qadir et al., 2020). Along similar lines, the current study observed a dominance of 41.05% by oxygenated monoterpenes in AgLEO. The predominant terpenes in AgLEO, eucalyptol, and (–)-terpinen-4-ol exhibit antibacterial, antifungal, and insecticidal effects, making them promising green pesticide candidates. Their primary mechanism of action often targets the pathogenic cell membranes, leading to structural and functional disruption (Połeć et al., 2019). This also supports a similar mechanism of AgLEO, as evidenced in **Figures 3**, **4**, showing hyphal structure disruption in *S. globosa*.

Fungal resistance against routine antifungal drugs broadly arises through target-site mutations, efflux pump overexpression, and genetic adaptations. The emergence of *Sporothrix* species with *in vitro* antifungal resistance owing to potential mechanisms including melanin production, genetic diversity, and mutations in cytochrome P450 enzymes has been well documented (Waller et al., 2021). In *S. globosa*, intrinsic tolerance is further reinforced by low genetic variability and adaptive selection. In our previous work, we demonstrated itraconazole resistance in this strain, further highlighting its ability to withstand frontline antifungal therapy. This highlights the need to unravel resistance pathways and identify novel therapeutic targets for human and veterinary applications.

The 4-h exposure of AgLEO at and above 0.25× MIC_50_ sufficiently induced an oxidative burst within the *S. globosa* yeast cells (**Figure 2**). Dysregulated intracellular redox homeostasis damages cellular functions and macromolecule levels, including lipids, proteins, and nucleic acids, and ultimately leads to cell dysfunction and death. These observations are consistent with established studies demonstrating that ROS generation in *S. globosa* is a downstream consequence of the stress response toward drug-mediated disruption of target processes. The ROS accumulation and depolarization of the mitochondrial membrane are vital in the apoptosis of fungi (Li and Du, 2024; Li et al., 2024). Interestingly, the comparator drug Amp B also elicited intracellular oxidative burst, an observation that has not been demonstrated previously in *S. globosa*. However, several reports have affiliated Amp B to oxidative burst in pathogenic yeasts of genus, *Candida* spp. and *Cryptococcus* spp (Mesa-Arango et al., 2014). Given that the classical antifungal action of Amp B involves binding to membrane ergosterol and forming pores, we subsequently examined membrane integrity in *S. globosa* post-AgLEO treatment (Gray et al., 2012). The patchy, uneven staining of CFW revealed deformed, distorted, and aggregated hyphae upon AgLEO treatment, reflecting a plausible cell membrane and structural disruption (**Figure 3**). Consistently, ergosterol quantification showed reduced levels, supporting that both oxidative stress and ergosterol depletion converge to destabilize cellular functions and the membrane architecture in *S. globosa*, thereby impairing the survival of yeast and fungal hyphal (**Figure 4**). Biofilms enhance fungal persistence by providing structural protection, reducing drug penetration, and conferring resistance to antifungals and host defenses. Disrupting biofilm formation weakens virulence, restores drug susceptibility, and improves treatment outcomes in sporotrichosis, making it a key therapeutic strategy. Biofilms in *Sporothrix* spp. are composed of yeast, hyphae, and extracellular matrix enriched with polysaccharides, proteins, and DNA, which enhance antifungal resistance and persistence. Their formation is linked to virulence and therapeutic failure, as biofilm-associated cells are less susceptible to antifungals, like itraconazole and Amp B. The observed disruption of biofilms by ALEO suggests interference with extracellular matrix stability, thereby reducing fungal resilience (**Figure 5**).

## 5 Conclusion

Collectively, these findings establish AgLEO as a promising antifungal botanical agent against *S. globosa*, exerting effects through ROS induction, structural disruption, ergosterol depletion, and inhibition of biofilm formation. By targeting both planktonic and biofilm-associated cells, both at the yeast and hyphae level, AgLEO offers therapeutic potential for managing sporotrichosis, particularly where conventional antifungals show limited efficacy (**Graphical Abstract**). Sporotrichosis is a neglected mycosis with rising antifungal resistance, highlighting the unmet need for innovative therapies.

## Data Availability

The original contributions presented in the study are included in the article/supplementary material. Further inquiries can be directed to the corresponding author.
